# Investigating the effectiveness of incorporating a stepped care approach into electronically delivered CBT for depression

**DOI:** 10.1192/j.eurpsy.2023.1030

**Published:** 2023-07-19

**Authors:** N. Alavi, M. Omrani, J. Jagayat, A. Shirazi, A. Kumar, A. Pannu, Y. Shao

**Affiliations:** 1 Psychiatry, Queen’s University, Kingston, Canada; 2 Psychiatry, OPTT inc, New York, United States; 3 neuroscience, Queen’s university, Kingston; 4 OPTT inc, Toronto; 5 Queen’s University, Kingston, Canada

## Abstract

**Introduction:**

Depression is a leading cause of disability, annually affecting up to 300 million people worldwide, yet fewer than one third of patients receive care. Cognitive behavioural therapy (CBT) is an effective treatment for depression, but there are barriers to access therapy. Electronic CBT (e-CBT) can address these barriers, but the digital format may reduce personalization and patient compliance. A balanced, hybrid model (i.e., combination of e-CBT & supervised care) could make therapy scalable and effective through a stepped-care model: a care model that begins treatment with the least resource intensive, yet effective, method while slowly ‘stepping up’ to intensive care based on patients’ needs.

**Objectives:**

-To examine the efficacy of a stepped-care e-CBT model for depression through reduction in depressive symptoms.

-To develop a decision-making process that can effectively allocate the appropriate level of care for each patient.

**Methods:**

This is a single-blinded randomized controlled trial (RCT). Participants were randomized to either the e-CBT group (n = 53) or the e-CBT with stepped care group (n = 26). Both groups received a 12/13-weeks e-CBT program tailored to depression. The e-CBT program was provided through a secure online mental health clinic called the Online Psychotherapy Tool (OPTT). Participants read through the sessions and completed assignments related to each session. Each participant was designated a care provider who was a trained research assistant. Participants in the experimental group received extra interventions based on their standard questionnaire scores, and textual data.

**Results:**

Figure 1: The average PHQ-9 (A), QLESQ (B), and QIDS (C) scores pre-, mid-, and post- treatment for the e-CBT only (n = 53) and stepped care groups (n = 26).

* Depressive symptoms: PHQ-9 (Patient Health Questionnaire-9) & QIDS (Quick Inventory of Depressive Symptomatology)

* Quality of Life Measure: QLESQ (Quality of Life Enjoyment and Satisfaction Questionnaire – Short Form)

**Image:**

**Figure:**
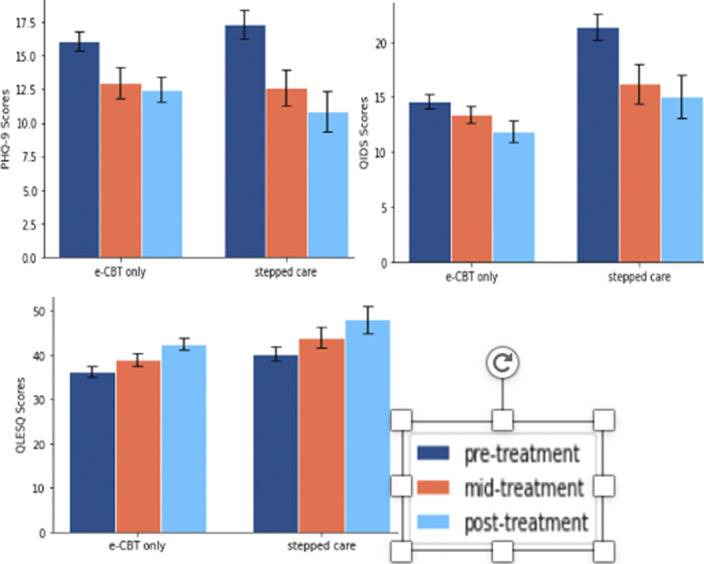

**Conclusions:**

Stepped care model can be reliable and effective method of delivering targeted care to future patients. Using this approach, the amount of care each patient receives is tailored to their needs, allowing for more efficient usage of scarce resources. This would also lower the general cost of care for each patient. By understanding the therapeutic needs of each patient, we can use these results to develop objective interventions and efficient algorithms to triage individuals. This technique could scale up care capacity without sacrificing the quality of care for each patient.

**Disclosure of Interest:**

N. Alavi Shareolder of: OPTT inc, M. Omrani Shareolder of: OPTT inc, J. Jagayat: None Declared, A. Shirazi: None Declared, A. Kumar: None Declared, A. Pannu: None Declared, Y. Shao: None Declared

